# Understanding the Structure–Function Relationship through 3D Imaging and Biomechanical Analysis: A Novel Methodological Approach Applied to Anterior Cruciate Ligaments

**DOI:** 10.3390/biomimetics9080477

**Published:** 2024-08-08

**Authors:** Marco Bontempi, Nicola Sancisi, Gregorio Marchiori, Michele Conconi, Matteo Berni, Giorgio Cassiolas, Gianluca Giavaresi, Annapaola Parrilli, Nicola Francesco Lopomo

**Affiliations:** 1 Complex Structure of Surgical Sciences and Technologies, IRCCS Istituto Ortopedico Rizzoli, Via di Barbiano 1/10, 40136 Bologna, Italy; 2 Department of Industrial Engineering, Alma Mater Studiorum—Università di Bologna, Viale del Risorgimento 2, 40136 Bologna, Italy; 3 Medical Technology Laboratory, IRCCS Istituto Ortopedico Rizzoli, Via di Barbiano 1/10, 40136 Bologna, Italy; 4 Movement Analysis Laboratory, IRCCS Istituto Ortopedico Rizzoli, Via di Barbiano 1/10, 40136 Bologna, Italy; 5 Center for X-ray Analytics, Swiss Federal Laboratories for Materials Science and Technology (Empa), Überlandstrasse 129, 8600 Dübendorf, Switzerland; 6 Department of Design, Politecnico di Milano, Via Durando, 20158 Milano, Italy

**Keywords:** anterior cruciate ligament (ACL), micro-CT, mathematical model, fiber microstructure

## Abstract

Understanding the microstructure of fibrous tissues, like ligaments, is crucial due to their nonlinear stress-strain behavior from unique fiber arrangements. This study introduces a new method to analyze the relationship between the microstructure and function of anterior cruciate ligaments (ACL). We tested the procedure on two ACL samples, one from a healthy individual and one from an osteoarthritis patient, using a custom tensioning device within a micro-CT scanner. The samples were stretched and scanned at various strain levels (namely 0%, 1%, 2%, 3%, 4%, 6%, 8%) to observe the effects of mechanical stress on the microstructure. The micro-CT images were processed to identify and map fibers, assessing their orientations and volume fractions. A probabilistic mathematical model was then proposed to relate the geometric and structural characteristics of the ACL to its mechanical properties, considering fiber orientation and thickness. Our feasibility test indicated differences in mechanical behavior, fiber orientation, and volume distribution between ligaments of different origins. These indicative results align with existing literature, validating the proposed methodology. However, further research is needed to confirm these preliminary observations. Overall, our comprehensive methodology shows promise for improving ACL diagnosis and treatment and for guiding the creation of tissue-engineered grafts that mimic the natural properties and microstructure of healthy tissue, thereby enhancing integration and performance in biomedical applications.

## 1. Introduction

Typically, the fundamental characteristics of a particular material are intrinsically linked to its structure within a hierarchical framework that varies with scale. In fact, when examining natural and artificial tissue materials, it is crucial to focus on their microstructure. This is especially important considering their multiphase, non-uniform, and frequently anisotropic composition at that level. For instance, the fibrous structures within the human musculoskeletal system, such as ligaments, exhibit very peculiar characteristics and behavior.

The Anterior Cruciate Ligament (ACL) is a key stabilizer in the knee, consisting primarily of dense connective tissue with parallel rows of fibroblasts and type I collagen [1]. It originates from the anterior and lateral aspects of the tibial spine and inserts obliquely upwards on the posteromedial side of the lateral femoral condyle [2]. The ACL functions dynamically, with groups of fibers adjusting tension throughout the knee’s range of motion, compensating for tensile stresses, and preventing excessive forward movement and rotational loads of the tibia [3]. Dense fibrous tissues such as the ACL exhibit nonlinear stress-strain characteristics. This is due to the uncrimping, reorientation, and tensioning of collagen fibers, which define their unique load-bearing properties [4]. The ACL, along with other ligaments, is characterized by a viscoelastic behavior in which stress relaxation and creep are phenomena essential in defining its specific properties [5]. Several studies indicate that stress relaxation is most pronounced during the first few seconds of testing. It gradually decreases over the next few minutes and finally stabilizes in about 1 h [6]. Creep, conversely, refers to the gradual stretching of the ligament under a constant load [7]. In order to describe its complex behavior, several modelling techniques were developed to study the mechanical properties of ACL and its response to different conditions [8,9]. However, constructing a single constitutive model that accurately captures the ACL behavior under different loading conditions remains challenging [10,11], especially considering the complex geometry of the ACL. Furthermore, it is worth noting that the inherent structural properties of the ligament can vary considerably between its various bundles [12]. Therefore, a combined understanding of the mechanical behavior and microstructural characteristics of ACL is essential. This is important to increase the general knowledge of its own physiology and pathological status, as well as to consider its peculiar properties in both ACL grafts and surgical reconstructions. Such understanding is crucial to avoid permanent changes in joint biomechanics. Measuring deformations on and within the ACL while characterizing its microstructure presents significant challenges. Recently, non-destructive imaging techniques have gained prominence. Magnetic resonance imaging (MRI) and ultrasound (US) have supported this goal but have important limitations when focusing on volumetric information at microstructural levels [13,14,15,16]. For studying tendons and ligaments, techniques like confocal, multiphoton, polarized light, scanning electron, or atomic force microscopies are required to resolve individual fibers and sub-fiber structures [17,18]. On the other hand, simple optical microscopy is ideal for 2D imaging the entire construct. Previous studies on ACL microstructure have provided information on only superficial aspects in limited areas using electron microscopy [19,20] or polarization imaging [12], and only in the latter case in combination with mechanical traction. In recent years, X-ray micro-computed tomography (micro-CT) has emerged as a powerful tool for studying the structure-function relationships of these materials in the intermediate length scale [21]. It provides high-resolution 3D images and 2D maps with volumetric resolutions approaching 1 μm [22,23,24,25,26,27], effectively revealing the natural volumetric fibrous arrangement and deformation. In this sense, phase-contrast enhanced synchrotron micro-tomography represented the State-of-the-Art for micro- and sub-micrometric analysis so far, but with limited accessibility [28,29]. Unlike conventional absorption-based X-ray analysis methods, phase contrast imaging exploits the phase shifts that X-rays undergo as they pass through different materials, potentially providing superior contrast for soft tissues and fibrous structures. However, it is essential to consider the radiation dose limitations associated with synchrotron sources and their limited and highly competitive access. While laboratory micro-CT sources typically deliver lower doses [30,31], synchrotron facilities can reach doses up to 35 kGy in ex vivo experiments, above which dose-dependent degradation in mechanical properties can be detected [32].

Laboratory-based micro-CT systems are much more accessible but suffer from low X-ray attenuation for soft tissues, resulting in poor contrast. Phase retrieval algorithms during reconstruction are gaining interest but remain insufficient for effectively identifying and separating fibers of fresh tissues. To overcome this problem, several researchers have turned to contrast agents designed to selectively bind specific tissues, thereby enhancing the contrast between the target tissue and its surrounding environment. Indeed, staining can enhance the X-ray contrast of the collagen fibers [33]. In this context, some studies have proposed the use of phosphotungstic acid (PTA) as a contrast agent of unloaded animal tissue [34,35]. A more recent study optimized this method for human tissue, ensuring that the original mechanical performance of the unstained tissue was not significantly altered [36].

This study aims to advance in this direction by employing contrast-enhanced laboratory-based X-ray micro-CT and mechanical tensile testing simultaneously. A comprehensive approach that includes biomechanical testing, reliable 3D imaging analysis, and numerical modeling is critical to unravel the relationship between biomechanical behavior and microstructural characteristics in ACL. Using the gathered data, we proposed a mathematical model that correlates the geometric and structural features of the tested tissue with its mechanical properties, accounting for factors like fiber orientation and thickness. The focus of the study is on the development of the approach to quantitatively demonstrate the relationship between fibrous arrangement and the mechanical behavior of the tissue. We applied this method to two human ACL specimens harvested from different sources as a proof of concept, making it a pilot study. This provides a methodological framework for future studies. With an adequate sample size, future research can offer conclusive interpretations about ligament/tendon alterations in pathologies or traumas. This could reveal target biomarkers for diagnosis and treatment, highlight the features that should be mimicked by tissue engineering, and be recovered by regenerative medicine.

## 2. Materials and Methods

### 2.1. Testing Protocol and Fibers Segmentation

Two specimens were analyzed; one was obtained from a cadaveric donor (age 40 years) with no knee pathologies (sample “A”), whereas the second one was harvested from a patient (age 69 years) undergoing total knee replacement due to primary osteoarthritis (sample “B”). They represent two opposite case studies whose differences can be analyzed and compared with our methodology. Both samples were frozen at −20 °C after harvesting. Each sample was thawed at room temperature and soaked in a contrast agent (PTA 2% in H_2_O) overnight to improve collagen fiber visualization with micro-CT. The protocol was approved by the local Ethics Committee and patient consent (Rizzoli Orthopedic Institute Committee, protocol “TISS-KNEE”, n. 8425, Bologna, Italy).

**Figure 1:**
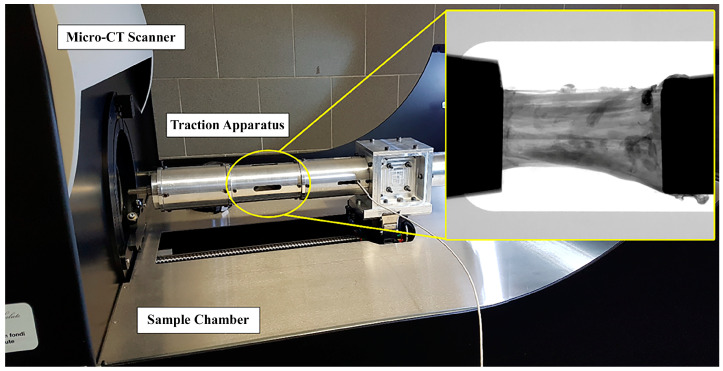
Integration of the traction apparatus into the micro-CT. The traction chamber is circled in yellow; a representative radiograph of the ligament tensioned between the clamps is included.

The two ACL specimens were prepared by caliper and cutter to present about the same length (20 mm) and cross-section area (34 mm^2^). To assess the effect of mechanical stress on the microstructure, both specimens were tensioned and scanned by micro-CT at different increasing levels of strain (i.e., ε = 0%, 1%, 2%, 3%, 4%, 6%, 8%). At each strain step, micro-CT scanning was performed after a 12-min wait to relax the sample. This duration was tested and found sufficient to complete stress relaxation in this context [36]. Each corresponding image acquisition lasted 14 min. Consequentially, the entire experiment on each sample lasted a total of 3 and a half hours. Specimens were kept wet by opening the micro-CT apparatus and moistening them with saline solution before each strain-scan step. A custom-made tensioning apparatus was designed to fit into the commercial micro-tomography (micro-CT) system Skyscan 1176 (Bruker, Kontich, Belgium). The device (Figure 1) consisted of a cylindrical chamber where the sample is maintained in position and elongated by two clamps with a manually operated threaded rod and a 1 kN load cell (1 N resolution; Burster Italia, Curno, BG, Italy), whose signal was acquired and interfaced to a personal computer. The structural parts of the tensioning device were made of an aluminum-zinc alloy, Ergal. The selection of Ergal over other alternatives such as Polyvinyl Chloride, Borosilicate glass, or stainless steel is based on its high machinability (tube thickness: 0.5 mm), good mechanical resistance (maximum testing force: 1000 N), and low X-ray attenuation (Ergal density: 2880 kg/m³). The thickness was chosen to withstand the maximum testing force and ensure reduced device compliance, with the maximum difference between the specimen’s nominal deformation and the effective one being below 0.2%. Using the Ergal tensioning device minimized the X-ray attenuation of the background (~14%), evaluated on planar images as the variation of the gray value along the device’s transverse section. Clamping was obtained with the aid of metal grips. Each clamp is composed of two flat surfaces covered by sandpaper and tightened at each ligament end by a screw. Full details on the testing protocol are described in [36].

Imaging acquisition was carried out by setting source voltage and current at 50 kV and 500 μA, respectively; the nominal resolution of the images was set at 9 μm (pixel size). Exposure time was 900 ms, and a total of 655 projections were acquired over 180° rotation. Source-to-Object Distance (SOD) and the Source-to-Detector Distance (SDD) were set to 124.201 mm and 174.045 mm, respectively. The field of view was 36 mm in width and 24 mm in height. Camera pixel binning was not applied (scan images 2672 × 4000 pixels).

The micro-CT datasets were then processed and analyzed. First, a region of interest (ROI) was defined on the central portion of the tensioned sample to exclude metal artifacts due to the clamps. The ROI was 4 mm long, starting from 1 mm above the fixed clamp.

**Figure 2:**
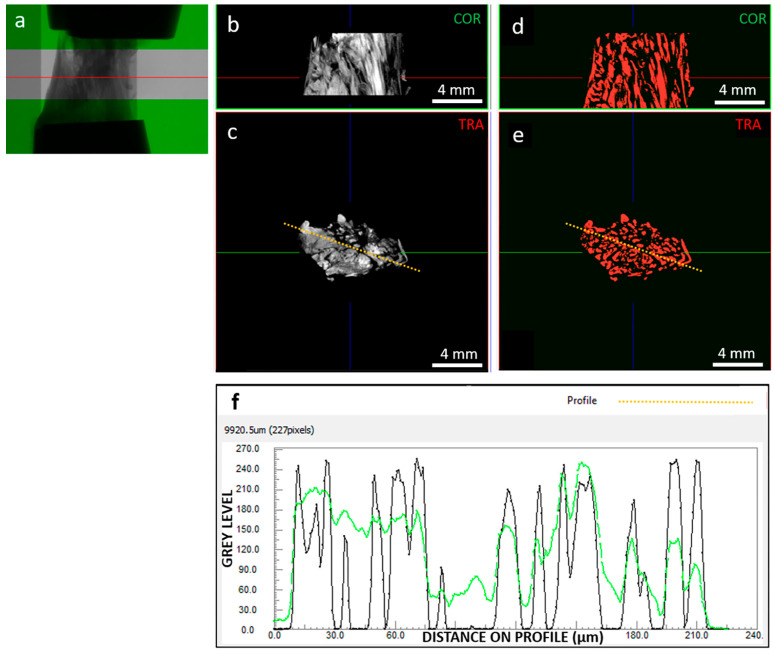
As an example, (**a**) radiography of clamped sample “A” at 0% strain highlighting out-of-green ROI; (**b**) coronal and (**c**) transversal sections of the grey level reconstructed image dataset, with corresponding coronal (**d**) and transversal (**e**) segmented dataset; (**f**) contrast profiles, in green for the orange dotted line on the grey scale image (**c**) and in black for the orange dotted line on the segmented image (**e**). Segmentation was able to resolve signal peaks corresponding to collagen fibers; in the example section, segmented objects (i.e., fibers) show an average thickness of 111 µm. The red line in images (**a**,**b**,**d**) indicates the transversal plane; the green line in images (**c**,**e**) indicates the coronal plane; the blue line in images (**b**–**e**) indicates the sagittal plane; the orange-dotted line in images (**c**,**d**) indicates the path along which the image contrast is profiled (**f**).

Basic assessment focused on image quality (Figure 2), which strongly impacts image processing for extracting tissue microstructural information. We investigated the signal (collagenous fibers) to noise (background and non-fibrous matrix) ratio. Images were reconstructed with 0–255 gray levels, and while the background was close to 0, the non-fibrous matrix signal was only slightly lower than the target fibrous signal. In addition, the spatial resolution was 9 µm, which limited the ability to resolve the hierarchical structure of the tissue down to fiber bundles [37]. Consequently, the internal tissue contrast was sufficient to visualize fibers but not to track them for segmentation purposes. This was achieved through developed image processing techniques, including local thresholding and morphological operations.

The grey-level image dataset in the ROI was binarized using the Niblack local thresholding algorithm (ImageJ software v. 1.53v [38,39]), which is one of the most used methods for dynamic image segmentation [40]. The individual objects corresponding to single identified fibers were separated using a combination of watershed, distance map, and H-Maxima methods with Avizo software v.2021.2 (Thermo Fisher Scientific, Waltham, MA USA). For each object/fiber, the mean cross-sectional area, volume, orientation in the transverse plane (φ°), and orientation with respect to the longitudinal, i.e., loading axis (θ°) was calculated using CTAn software v.1.20.3.0 (Bruker, Kontich, Belgium). In this way, a population of objects with their relative population of areas, volumes, and orientations was obtained for each strain level scanned. Objects with θ = 90° and φ = 0°, i.e., “collapsed” on the transverse plane, were considered artifacts and excluded from the analysis. The others were considered actual fibers and were included in the mechanical modeling and analysis. 

### 2.2. Analysis of Mechanics-Structure Relation

The design and implementation of the traction apparatus of the tensioning protocol and of the micro-CT imaging originate from [36]. However, the structural data provided by micro-CT offer limited information about the ligament’s mechanical response under load. To bridge this gap, a mathematical model was developed to relate the microstructure of the tested bundles to their respective mechanical responses. Thus, the present study describes the integration of imaging and mechanical information, revealing structure by image analysis and then modelling the mechanics-structure relation. Specifically, the data obtained from micro-CT images and those obtained from mechanical tensile tests were merged to correlate the geometric/structural properties of the bundle with its mechanical properties. For this purpose, a mathematical model of the bundle was defined that took into account the mechanical response of the bundle (stress/strain) and the spatial orientation and thickness of the fibers.

The model was structured according to the probabilistic model described by various authors. The starting model was the probabilistic model described by Bontempi et al. [41]:

(1) $$F\left(L\right)=N_{0}\int_{L_{0}}^{L}R\left(x\right)f\left(L-x\right)dx$$

where *F*(*L*) is the force recovered by the bundle when subjected to a length *L*, *L*_0_ is the rest length (*F*(*L*_0_) = 0), *N*_0_ is the number of fibers, *R*(.) is the recruitment distribution of the fibers participating in the force, *f*(.) is the force of a single fiber in the bundle in response to elongation.

Equation (1) was rewritten in terms of stress/strain. Stress was defined as the force divided by the total cross-sectional area of the bundle (σ = *F*/*A_tot_*), while strain was defined as the ratio of bundle length to resting length (*λ* = *L*/*L*_0_).

Replacing into Equation (1), the model in terms of stress/strain becomes:

(2) $$\sigma \left(\lambda \right)=\frac{N_{0}}{A_{tot}}\int_{1}^{\lambda }R(\xi )f\left(\lambda -\xi \right)d\xi$$

**Figure 3:**
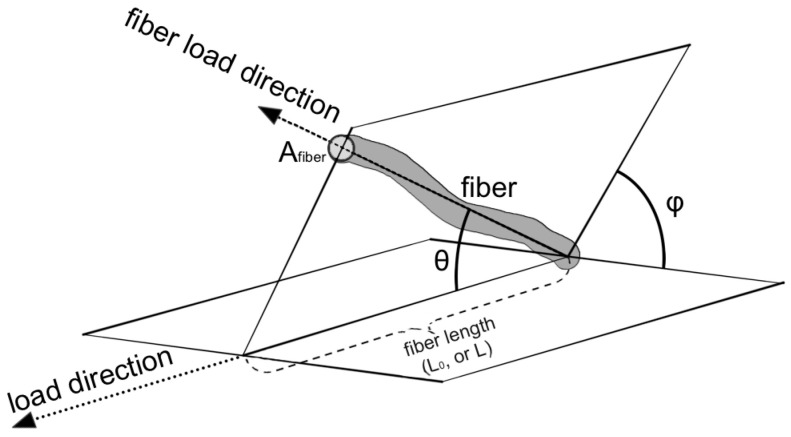
Model geometry representation. The picture shows the parameters used in the model: orientation of the fiber (θ, φ angles), the length of the fiber (L_0_, L), and the cross area (A_fiber_) of the fiber.

Each fiber within the bundle has its own orientation in space (Figure 3).

The fibers were considered uniformly oriented with respect to the plane perpendicular to the load (angle *φ*), and their orientation in the direction of the load was evaluated (angle *θ*), as suggested by Hurschler et al. [42]. The fibers within the bundle are organized to transmit force along the direction of strain. They can be represented by an angular distribution $\left(\int_{0}^{2\pi }\int_{0}^{\frac{\pi }{2}}P\left(\theta \right)\sin\theta d\theta d\varphi =1\right)$. Considering Equation (2) as multiplied by 1 and substituting with the normalization of the angular distribution, the angular dependence of the model is made explicit:

(3) $$\sigma \left(\lambda \right)=2\pi \frac{N_{0}}{A_{tot}}\int_{0}^{\frac{\pi }{2}}\int_{1}^{\lambda }R(\xi )P\left(\theta ,\xi \right)f\left(\lambda -\xi \right)\sin\theta d\xi d\theta$$

where the constant 2*π* accounts for uniform distribution with respect to angle *φ*, while *P*(.) is the fiber distribution along the strain direction. In the most general case, *P*(.) also depends on strain to account for the fact that fibers change orientation as strain changes.

Single fiber force (*f*) can be rewritten in terms of single fiber stress by applying the definition used earlier. In this case, called *s*(.) the fiber stress and *A*(.) the fiber cross section, the single fiber force can be written as *f*(.) = *A*(.)*s*(.). Cross sections can be expressed as a function of the volume of fibers and bundles. Hence, we have that *V_tot_ = A_tot_L*_0_*λ* and *V*(.) *= A*(.)*L*_0_*λ*. Substituting in Equation (3), the ratio *A*(.)*/A_tot_ = V*(.)*/V_tot_*. The latter expression is the volumetric fraction of the fibers. Denoting it by *ν*(.) and substituting in equation 3, the model becomes:

(4) $$\sigma \left(\lambda \right)=2\pi N_{0}\int_{0}^{\frac{\pi }{2}}\int_{1}^{\lambda }R(\xi )P\left(\theta ,\xi \right)s\left(\lambda -\xi \right)\nu \left(\xi ,\;\theta \right)\sin\theta d\xi d\theta$$

where *ν* was expressed in the most general form, i.e., dependent on both orientation and strain.

The last step was to define the fiber’s orientation within the model. For simplicity, following the results of Epel et al. [43] and Shen et al. [44], the single fiber stress was defined as a linear function of strain, and the constant of proportionality is the Young’s modulus of fibers (*E_f_*). Then the orientations were included (*s*(.) *= E_f_* (./cos *θ*)), and the final equation of the model is:

(5) $$\sigma \left(\lambda \right)=2\pi N_{0}E_{f}\int_{0}^{\frac{\pi }{2}}\int_{\frac{1}{\cos\theta }}^{\frac{\lambda }{\cos\theta }}R\left(\xi \right)P\left(\theta ,\;\xi \right)\left(\frac{\lambda }{\cos\theta }-\xi \right)\nu \left(\xi ,\;\theta \right)\cos^{2}\theta \sin\theta d\xi d\theta$$

where, to simplify the notation, the substitution $\frac{\varepsilon }{\cos\theta }\to \varepsilon$ was made.

The *P*(.) distribution can be determined experimentally, but not the *R*(.). For this reason, it was modelled by following Hurschler et al. [42], using the Weibull distribution:

(6) $$\left(\xi \right)=\frac{\beta }{\delta }{\left(\frac{\xi -\gamma }{\delta }\right)}^{\beta -1}e^{-{\left(\frac{\xi -\gamma }{\delta }\right)}^{\beta }}$$

where parameters *β* and *δ* are characteristic parameters of the distribution, *λ* is the strain, *γ* is the initial strain, and, in this case, was set to one (*γ* = 1). From a physical point of view, the δ parameter (scale) is related to the variation of fiber recruitment lengths, while the *β* parameter (shape) is related to the structure of recruitment lengths: *β* < 1 implies a high number of fibers recruited at the beginning of the stretch and a steady decrease as the stretch progresses. At the limit, when *β* = 1, there is a constant fiber recruitment rate. Then, when *β* > 1, the number of fibers recruited increases as the stretch increases to a maximum and then decreases. The model developed (Equation (2)) was used to estimate the mechanical and structural characteristics of the fibers composing the ligaments analyzed by micro-CT. First of all, the data obtained from the micro-CT images were filtered by eliminating degenerate objects that make no contribution to the load (e.g., zero volumes or orientations orthogonal to the load). Then, interpolations were made on the data of the ligament indicated as sample “A” and of that indicated as sample “B”. The interpolations were implemented in MATLAB^®^ (R2018b, The MathWorks Inc., Natick, MA, USA) using a nonlinear minimization algorithm. The parameters taken into consideration were *E_f_*; *β* (shape) and *δ* (scale) from Equation (3). The cost function was implemented as the mean square deviation between the model in Equation (2) and the experimental data. The computed parameters were compared to findings in the relevant literature to provide preliminary validation, considering their extent and dependence on the pathophysiological condition of the tissue.

## 3. Results

### 3.1. Fibers Segmentation

**Figure 4:**
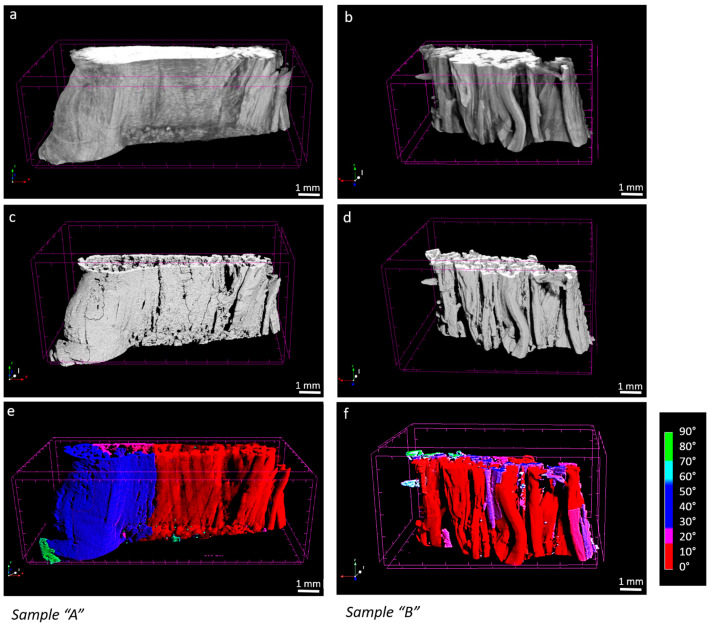
4% strain, sample “A” and sample “B” micro-CT original image (**a**,**b**), processed image (**c**,**d**), fibers mapped by orientation ((**e**,**f**), where red is 0° and blue 90° respect to the loading, axis).

In the micro-CT images, sample “A” appeared to be larger in volume and qualitatively denser in the periphery compared to sample “B” (Figure 4a,b). This suggests that, as expected, tissue composition and the extracellular matrix conditions influence the degree of contrast agent diffusion into the sample. Such differences between healthy “A” and pathological “B” tissues can potentially affect the segmentation and mapping of fibers during image processing (Figure 4c–f). Nevertheless, the segmentation approach we applied, which is based on a local thresholding algorithm, remained consistent in both inter-samples and intra-samples between strains. In sample “A”, the number of objects considered as fibers showed a coefficient of variation on strains of 3%, while in sample “B”, that percentage was 5%. In sample “A”, fibers represented the 75% of the segmented objects, whereas in the sample “B”, the 80%. Objects considered as artifacts had a volume below just 15.8 × 10^−5^ mm^3^ and 14.7 × 10^−5^ mm^3^ for “A” and “B”, respectively.

### 3.2. Analysis of Mechanics-Structure Relation

**Figure 5:**
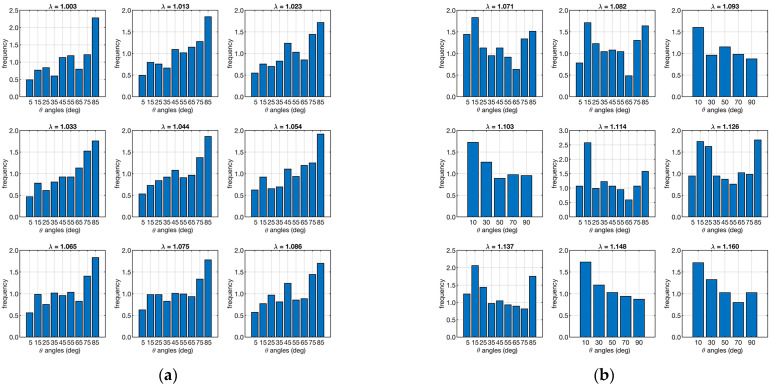
Angular distribution of the number of fibers along the loading direction. The left plot (**a**) refers to the ligament sample “A”, and the right plot (**b**) is to sample “B”. Each set of 9 plots represents the angular distribution in relation to λ (shown as a small title of each plot).

Micro-CT performed on the test ligaments allowed the *P*(*θ*, *λ*) and ν(*θ*, *λ*) distributions to be evaluated. Figure 5 shows the distributions of fiber orientations for each strain in the two cases analyzed.

The angular distribution of fibers in a ligament, such as the ACL, is crucial for understanding its mechanical behavior. As the ligament is stretched (increasing λ), the fibers within the ligament reorient themselves to align more with the direction of the applied load. This reorientation is necessary to bear the increased load efficiently, minimizing the risk of injury or failure. The mathematical model incorporates *P*(*θ*, *λ*) to predict how the mechanical properties of the ligament change with different levels of stretch. The histograms of Figure 5 show that in case “A”, they tend to orient toward large angles and maintain this orientation during strain. In contrast, the fibers in sample “B” show an opposite trend. To quantify this difference, the skewness of the various distributions was analyzed, and the results are shown in Table 1.

The sample “A” has negative skewness, while the sample “B” has positive skewness. This confirms that the fiber orientation of the first ligament contains more objects oriented in different directions than the loading direction. In contrast, the second ligament has more fibers in the direction of loading.

**Figure 6:**
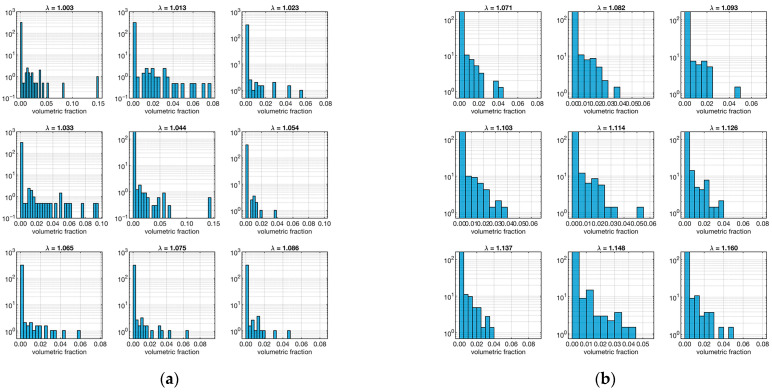
Distribution fiber volume fraction. The left plot (**a**) refers to the ligament sample “A”, and the right plot (**b**) is to sample “B”. Each set of 9 plots represents the distribution in relation to *λ* (shown as a small title of each plot).

The other important model parameter that was evaluated from the micro-CT data is the volume distribution of segmented fibers. The relative distributions as a function of the strain (*λ*) are shown in Figure 6.

In this case, both samples show a majority of low-volume fibers in all strains. As before, to quantify this aspect, the skewness of the distributions at various strain levels was calculated, as shown in Table 2.

As expected, all skewness was positive, confirming the trend. However, an interesting fact emerged: sample “A” tends to have higher skewness values than sample “B”. This means that the fibers in sample “B” tend to be bulkier than those in sample “A”.

**Figure 7:**
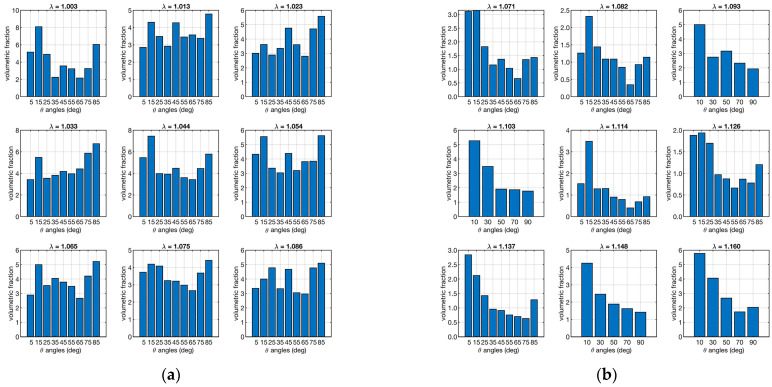
Distribution fiber volume fractions as a function of orientation. The left plot (**a**) refers to the ligament sample “A”, and the right plot (**b**) is to sample “B”. Each set of 9 plots represents the distribution in relation to *λ* (shown as a small title of each plot).

According to Equation (5), the presence of small fibers can have a significant impact on the load response by the applied load. Therefore, the combined data of orientation and strain in the distribution of volumetric fractions were evaluated in order to assess the effectiveness of the response of bulky and small fibers. Figure 7 shows the distribution of volumetric fractions as a function of orientations and strain (*ν*(*θ*, *φ*)).

Figure 7 shows that there is a more homogeneous distribution of volumetric fractions in sample “A” than in sample “B”, which, in contrast, shows more bulky fibers in the loading direction.

The last parameters that were evaluated from the experimental data, using the model in Equation (5), were Young’s modulus (*E_f_*) and the parameters of scale (*δ*) and shape (*β*) of the recruitment distributions (*R*(*λ*)) of the two samples. The results of the optimizations are shown in Table 3.

**Figure 8:**
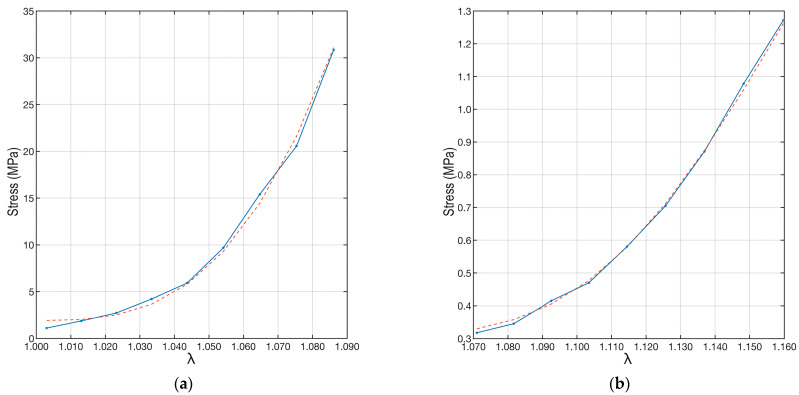
Plots of the experimental data and their interpolations. The red dotted line refers to the interpolation made by applying Equation (6). The left plot (**a**) refers to the “A” ligament, and the right plot (**b**) is to the “B” ligament.

Once all model parameters were determined, the experimental data were interpolated using Equation (5) and substituting the parameter values and distributions just described. The result is shown in Figure 8. The proposed interpolation perfectly follows the trend of the experimental data results from the load tests. 

To quantify the correctness of the interpolation, the coefficient of determination (R^2^) [45] was used. This coefficient can take values between 0 and 1, where 0 indicates no fit between the model and data, while 1 implies a perfect fit between the model and data. In the case of samples “A” and “B”, the coefficient of determination was R^2^_A_ = 0.996, while R^2^_B_ = 0.998. This shows the excellent agreement between the fit and the data.

**Figure 9:**
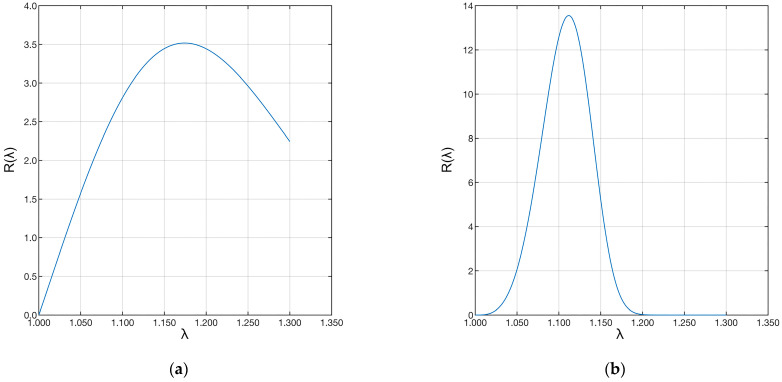
Plot of the Weibull distributions associated with the data. The left plot (**a**) refers to the “A” ligament, and the right plot (**b**) to the “B” ligament.

Finally, the shape (*β*) and scale (*δ*) parameters were used to evaluate the distribution of fiber recruitment in the two samples. The two distributions are shown in Figure 9.

Sample “A” shows a much longer distribution than sample “B”. This difference shows that the first ligament has fibers with much longer recruitment lengths than the second ligament. Therefore, few fibers were recruited during strain, and the recruitment process was still in progress. The same applies to the fibers of the pathological ligament. However, in this case, many more fibers were recruited, shortening the toe region of the pathological ligament. In both cases, the ligaments were still in the toe region.

## 4. Discussion

In our study, we developed a processing procedure to investigate changes in the volumetric microstructure of fibrous tissues subjected to progressive tensile loads. This procedure integrates micro-CT imaging at different loading conditions with a specifically implemented numerical modelling. The new probabilistic model links geometrical and analytical characterization to explain the effect of 3D microstructural properties of the ligament on its comprehensive mechanical behavior. The integration of biomechanical testing, imaging analysis, and modelling is considered to provide new insights and advance our understanding of fibrous microstructure and function, paving the way for more informed and effective treatment and regeneration strategies. To test the feasibility of the developed methodology and to show its scientific potential, we applied the procedure to two ligament samples: one from a healthy donor and one from a patient with pathological knee conditions (i.e., OA). We then compared the results with the literature.

From an image processing perspective, the study of Maksimcuka et al. [21] on X-ray tomographic imaging of electrospun fiber constructs for tissue engineering provided important guidelines for our approach. They emphasized segmenting individual “fibers” and tracking their changes in arrangement due to tensile deformation, including reorientation and fiber thinning. Our study adopted a similar approach but with three key differences. First, our study is based on biological rather than artificial fibrous materials, presenting a challenge for X-ray tomographic contrast. Additionally, segmentation of collagen “fibers” is more difficult than for artificial fibers due to the presence of a non-fibrous extracellular matrix between the fibers [19]. Second, we performed a 4D analysis by imaging the same sample at multiple time strains, while Maksimcuka et al. [21] realized a 3D analysis on different samples, each at a single strain. Third, our aim was to reveal the constitutive property of “fibers”, such as the elastic modulus, in addition to observing the structural changes during deformation.

The mathematical model presented describes the fibrous response as a function of load and elongation (Equation (5)), taking into account the fiber recruitment distribution, fiber orientation, and fiber volume ratio. This model represents our results of stress as a function of strain. Its application to ligaments obtained from healthy and pathological subjects allowed a first validation of the overall procedure. We interpolated the experimental data obtained from tensile tests and image reconstructions with micro-CT using the model described in equations 5 and 6. The calculations showed that a wide combination of parameters can be used to interpolate the data. In particular, the linear dependence on Young’s modulus (*E_f_*) made it possible to correct the final curve to fit the experimental data. 

The results aligned with the literature values for *E_f_*, and an excellent agreement was achieved for both ACL samples as quantified by the coefficient of determination. Furthermore, the computed recruitment distribution and the calculated *E_f_* showed a good compromise. The fiber angular distribution (Figure 5, Table 1) showed large differences at different strains for the two specimens. In particular, the bundle from the healthy joint (sample “A”) showed more fibers aligned with the loading direction, while the bundle from the pathological joint (sample “B”) showed an opposite distribution. Although similar, the volume fractions were more equally distributed across the orientation angles in the healthy sample compared to the pathological one (Figure 7). The healthy sample also presented fibers with major recruitment lengths, indicating ongoing recruitment during strain. The pathological sample had a shorter stress-strain toe region due to more fibers being recruited. Finally, the fiber Young’s modulus (Table 3, *E_f_*) showed very different values for the two ligaments, differing by two orders of magnitude.

The recent study by Peters et al. [46] is a good reference for comparing our results. In our study, sample “A” came from a healthy young donor, and sample “B” came from an old OA donor. Load, stress, and Young’s modulus values for sample “A” were within the range of healthy young ACLs [46], while sample “B” showed 10 times lower load, 30 times lower stress, and 200 times lower modulus. In Peters et al. [46], even if they cannot statistically derive the combined effect of age and OA on ACL stress/modulus, they reported a factor of 10 difference between healthy young and OA old samples. Similarly, Woo et al. [47] observed a decrease in mechanical parameters by one order of magnitude from middle-aged to older subjects, factoring in the deteriorating effect of pathology (i.e., osteoarthritis) in addition to age [48]. It must be acknowledged that in our study, sample “B” appeared particularly loose and soft prior to testing, and despite efforts to limit slippage with sandpaper, it could not be fully excluded, lowering the specimen’s resistance to deformation. Preservation and clamping of the ligament attachment bone stocks could upgrade the experimental procedure. However, the method for calculating Young’s modulus introduced by this study focuses on the actual resistance to deformation of collagen fibers, potentially revealing greater differences that are intrinsic to the lower micrometric tissue scale. Thus, overall, the developed methodology can be considered valid and has elements of innovation.

The practical use of our methodological approach lies in its potential to advance the understanding of the microstructural properties and biomechanics of dense fibrous tissues, their pathophysiology, and consequently guide treatment. For example, in the context of ligament and tendon reconstruction, our methodology can be used to guide the design and selection of different graft options by analyzing their microstructural and mechanical properties. This is particularly important in biomedical engineering, where designing grafts that closely mimic the properties of natural tissues is essential for their successful outcomes. The results in Table 3 and Figure 8 indicate that the ligament from the healthy joint (sample “A”) can offer a progressive but still bold restriction to motion. At low deformation levels, this is related to the strong properties of the fibers, while at higher strain levels, fiber recruitment and rearrangement could increase their role. In other words, the better performance of one sample in resisting continuous traction seems to be related not to a more favorable fibrous arrangement (recruitment and anisotropy) at the microscale. Instead, it is related to higher mechanical properties of the fibers, depending on their composition and structure at the lower hierarchical levels, which are not visible by our imaging micro-CT protocol. Various studies support our findings [48,49,50], indicating that structural/compositional alterations responsible for lower mechanical properties should generally be sought at a lower, micrometer-or-less spatial scale (e.g., hierarchically, from fibers to fibrils).

This study lacks statistical significance in comparing a single “healthy” ligament with a single “pathological” ligament. However, the aim was to develop and present a mathematical model that accounts for the mechanical responses (stress/strain) of the bundle as well as the spatial orientation and thickness of the fibers. In addition to presenting the experimental facets and the setup, as conducted preliminarily in previous work [28], this study emphasizes the theoretical framework to enhance understanding of the underlying mechanics. The processing of micro-CT images is fundamental as it provides the microstructural input to the constitutive model. However, it still lacks specific validation, which is the major limitation of this study. Future work should focus on this, for example, by performing histomorphometry in parallel [51]. Moreover, while much effort has been made to enhance the micro-CT contrast of collagenous fibers in sample preparation, there is still significant room for improvement, particularly in image processing. Work is currently in progress to address these areas. 

## 5. Conclusions

This study developed a comprehensive methodology to investigate the volumetric microstructure and mechanical behavior of ACL fibrous tissue under progressive tensile loading. To test the feasibility of our method, we used two different ACL samples, one from a healthy individual and one from an osteoarthritis patient.

By integrating X-ray micro-CT imaging, mechanical tensile testing, and a new probabilistic numerical model that considers the internal structure of the ligament, we analyzed the possibility of correlating the geometric and structural features of the tissue with its mechanical properties. The particular structure of the model made it possible to provide an answer that consistently accounted for all the parameters involved. In particular, there are several combinations of parameters that fit excellently with the experimental data. This fact, although it may seem disadvantageous, could be used to tailor further experimental evaluations to different ligaments and different conditions. 

The preliminary results indicate that the healthy ACL sample exhibits superior mechanical properties, with fibers having a more favorable arrangement and longer recruitment lengths compared to the pathological sample. The study also highlighted the significant differences in Young’s modulus between the two samples, which is consistent with existing literature on age and pathology-related changes in ligament properties.

Despite the limitations of the study, including a small sample size and the need for further validation through histomorphometry, the methodology developed provides a valuable framework for future research. The proposed approach has the potential to advance the understanding of the microstructural properties and biomechanics of dense fibrous tissues and their pathophysiology, and consequently, it can best guide treatment. In the case of ligaments and tendons, it can be used to assess graft options for reconstruction on tissue engineering solutions. Future efforts will focus on improving micro-CT contrast and image processing techniques to further refine the model and its applications.

## Figures and Tables

**Table 1:** Values of skewness of the corresponding angular distributions shown in Figure 5.

Sample “A”			Sample “B”		
−0.36	−0.35	−0.32	0.21	0.11	0.12
−0.41	−0.30	−0.35	0.22	0.34	0.18
−0.23	−0.21	−0.23	0.28	0.24	0.26

**Table 2:** Values of skewness of the corresponding volumetric ratio distributions shown in Figure 6.

Sample “A”			Sample “B”		
11.03	6.46	6.15	4.31	3.27	3.73
7.45	10.21	7.42	3.57	3.47	3.80
6.42	6.56	6.69	4.45	2.86	4.04

**Table 3:** Optimization results on model parameters for ligaments “A” and “B”.

	Sample “A”	Sample “B”
*E_f_* (MPa)	100.0017	0.5243
*β*	2.0143	4.2552
*δ*	0.2450	0.1190

## Data Availability

The raw data supporting the conclusions of this article will be made available by the authors upon request.

## References

[1] Dargel J., Gotter M., Mader K., Pennig D., Koebke J., Schmidt-Wiethoff R. (2007). Biomechanics of the anterior cruciate ligament and implications for surgical reconstruction. Strateg. Trauma Limb Reconstr..

[2] Parrilli A., Grassi A., Orellana F., Lolli R., Marchiori G., Berni M., Fini M., Lopomo N.F., Zaffagnini S. (2024). 3D visualization of the human anterior cruciate ligament combining micro-CT and histological analysis. Surg. Radiol. Anat..

[3] Domnick C., Raschke M.J., Herbort M. (2016). Biomechanics of the anterior cruciate ligament: Physiology, rupture and reconstruction techniques. World J. Orthop..

[4] Hirokawa S., Tsuruno R. (2000). Three-dimensional deformation and stress distribution in an analytical/computational model of the anterior cruciate ligament. J. Biomech..

[5] Dienst M., Burks R.T., Greis P.E. (2002). Anatomy and biomechanics of the anterior cruciate ligament. Orthop. Clin. N. Am..

[6] Pioletti D.P., Rakotomanana L.R. (2000). On the independence of time and strain effects in the stress relaxation of ligaments and tendons. J. Biomech..

[7] Frank C.B. (2004). Ligament structure, physiology and function. J. Musculoskelet. Neuronal Interact..

[8] Benos L., Stanev D., Spyrou L., Moustakas K., Tsaopoulos D.E. (2020). A Review on Finite Element Modeling and Simulation of the Anterior Cruciate Ligament Reconstruction. Front. Bioeng. Biotechnol..

[9] Knapp A., Williams L.N. (2022). Predicting the Effect of Localized ACL Damage on Neighbor Ligament Mechanics via Finite Element Modeling. Bioengineering.

[10] Luetkemeyer C.M., Scheven U., Estrada J.B., Arruda E.M. (2021). Constitutive modeling of the anterior cruciate ligament bundles and patellar tendon with full-field methods. J. Mech. Phys. Solids.

[11] Readioff R., Geraghty B., Comerford E., Elsheikh A. (2020). A full-field 3D digital image correlation and modelling technique to characterise anterior cruciate ligament mechanics ex vivo. Acta Biomater..

[12] Castile R.M., Skelley N.W., Babaei B., Brophy R.H., Lake S.P. (2016). Microstructural properties and mechanics vary between bundles of the human anterior cruciate ligament during stress-relaxation. J. Biomech..

[13] Zubrod C.J., Barrett M.F. (2007). Magnetic Resonance Imaging of Tendon and Ligament Injuries. Clin. Tech. Equine Pract..

[14] Robinson P. (2009). Sonography of common tendon injuries. AJR Am. J. Roentgenol..

[15] Chang A., Miller T.T. (2009). Imaging of tendons. Sports Health.

[16] Weinreb J.H., Sheth C., Apostolakos J., McCarthy M.B., Barden B., Cote M.P., Mazzocca A.D. (2014). Tendon structure, disease, and imaging. Muscles Ligaments Tendons J..

[17] Liu J., Xu M.Y., Wu J., Zhang H., Yang L., Lun D.X., Hu Y.C., Liu B. (2021). Picrosirius-Polarization Method for Collagen Fiber Detection in Tendons: A Mini-Review. Orthop. Surg..

[18] Puetzer J.L., Ma T., Sallent I., Gelmi A., Stevens M.M. (2021). Driving Hierarchical Collagen Fiber Formation for Functional Tendon, Ligament, and Meniscus Replacement. Biomaterials.

[19] Clark J.M., Sidles J.A. (1990). The interrelation of fiber bundles in the anterior cruciate ligament. J. Orthop. Res..

[20] Zhu J., Zhang X., Ma Y., Zhou C., Ao Y. (2012). Ultrastructural and morphological characteristics of human anterior cruciate ligament and hamstring tendons. Anat. Rec. (Hoboken).

[21] Maksimcuka J., Obata A., Sampson W.W., Blanc R., Gao C., Withers P.J., Tsigkou O., Kasuga T., Lee P.D., Poologasundarampillai G. (2017). X-ray Tomographic Imaging of Tensile Deformation Modes of Electrospun Biodegradable Polyester Fibers. Front. Mater..

[22] Zuncheddu D., Della Bella E., Schwab A., Petta D., Rocchitta G., Generelli S., Kurth F., Parrilli A., Verrier S., Rau J.V. (2021). Quality control methods in musculoskeletal tissue engineering: From imaging to biosensors. Bone Res..

[23] Grassi A., Dal Fabbro G., Fini M., Zaffagnini S., Parrilli A. (2021). Case Report: Anterior Cruciate Ligament Calcification in a Patient With Chondrocalcinosis: Micro-Computed Tomography Presentation. Front. Surg..

[24] Parrilli A., Giavaresi G., Ferrari A., Salamanna F., Desando G., Grigolo B., Martini L., Fini M. (2017). Subchondral bone response to injected adipose-derived stromal cells for treating osteoarthritis using an experimental rabbit model. Biotech. Histochem..

[25] Boerckel J.D., Mason D.E., McDermott A.M., Alsberg E. (2014). Microcomputed tomography: Approaches and applications in bioengineering. Stem Cell Res. Ther..

[26] Vásárhelyi L., Kónya Z., Kukovecz A., Vajtai R. (2020). Microcomputed tomography–based characterization of advanced materials: A review. Mater. Today Adv..

[27] Orellana F., Grassi A., Hlushchuk R., Wahl P., Nuss K.M., Neels A., Zaffagnini S., Parrilli A. (2024). Revealing the complexity of meniscus microvasculature through 3D visualization and analysis. Sci. Rep..

[28] Pierantoni M., Silva Barreto I., Hammerman M., Verhoeven L., Törnquist E., Novak V., Mokso R., Eliasson P., Isaksson H. (2021). A quality optimization approach to image Achilles tendon microstructure by phase-contrast enhanced synchrotron micro-tomography. Sci. Rep..

[29] Zhou Y., Hu J., Zhou J., Zeng Z., Cao Y., Wang Z., Chen C., Zheng C., Chen H., Lu H. (2018). Three-dimensional characterization of the microstructure in rabbit patella-patellar tendon interface using propagation phase-contrast synchrotron radiation microtomography. J. Synchrotron Radiat..

[30] Laperre K., Depypere M., van Gastel N., Torrekens S., Moermans K., Bogaerts R., Maes F., Carmeliet G. (2011). Development of micro-CT protocols for in vivo follow-up of mouse bone architecture without major radiation side effects. Bone.

[31] Pratt I.V., Belev G., Zhu N., Chapman L.D., Cooper D.M. (2015). In vivo imaging of rat cortical bone porosity by synchrotron phase contrast micro computed tomography. Phys. Med. Biol..

[32] Barth H.D., Zimmermann E.A., Schaible E., Tang S.Y., Alliston T., Ritchie R.O. (2011). Characterization of the effects of x-ray irradiation on the hierarchical structure and mechanical properties of human cortical bone. Biomaterials.

[33] Bushara F., Maglio M., Marchiori G., Giavaresi G., Signoroni A., Guerrini F., Lopomo N.F. (2023). Contrast-enhanced microtomography for volumetric analysis of microstructure in ligaments and tendons. J. Mech. Med. Biol..

[34] Balint R., Lowe T., Shearer T. (2016). Optimal Contrast Agent Staining of Ligaments and Tendons for X-Ray Computed Tomography. PLoS ONE.

[35] Shearer T., Rawson S., Castro S.J., Balint R., Bradley R.S., Lowe T., Vila-Comamala J., Lee P.D., Cartmell S.H. (2014). X-ray computed tomography of the anterior cruciate ligament and patellar tendon. Muscles Ligaments Tendons J..

[36] Marchiori G., Parrilli A., Sancisi N., Berni M., Conconi M., Luzi L., Cassiolas G., Zaffagnini S., Lopomo N.F. (2019). Integration of micro-CT and uniaxial loading to analyse the evolution of 3D microstructure under increasing strain: Application to the anterior cruciate ligament. Mater. Today Proc..

[37] Gohl K.L., Listrat A., Béchet D. (2014). Hierarchical mechanics of connective tissues: Integrating insights from nano to macroscopic studies. J. Biomed. Nanotechnol..

[38] Schneider C., Rasband W., Eliceiri K. (2012). NIH Image to ImageJ: 25 years of image analysis. Nat. Methods.

[39] Niblack W. (1986). An Introduction to Digital Image Processing.

[40] Korzynska A., Roszkowiak L., Lopez C., Bosch R., Witkowski L., Lejeune M. (2013). Validation of various adaptive threshold methods of segmentation applied to follicular lymphoma digital images stained with 3,3′-Diaminobenzidine&Haematoxylin. Diagn. Pathol..

[41] Bontempi M. (2009). Probabilistic model of ligaments and tendons: Quasistatic linear stretching. Phys. Rev. E Stat. Non. Soft Matter Phys..

[42] Hurschler C., Loitz-Ramage B., Vanderby R. (1997). A structurally based stress-stretch relationship for tendon and ligament. J. Biomech. Eng..

[43] Eppell S.J., Smith B.N., Kahn H., Ballarini R. (2006). Nano measurements with micro-devices: Mechanical properties of hydrated collagen fibrils. J. R. Soc. Interface.

[44] Shen Z.L., Dodge M.R., Kahn H., Ballarini R., Eppell S.J. (2008). Stress-strain experiments on individual collagen fibrils. Biophys. J..

[45] Magee L. (1990). R2 Measures Based on Wald and Likelihood Ratio Joint Significance Tests. Am. Stat..

[46] Peters A.E., Geraghty B., Bates K.T., Akhtar R., Readioff R., Comerford E. (2022). Ligament mechanics of ageing and osteoarthritic human knees. Front. Bioeng. Biotechnol..

[47] Woo S.L., Hollis J.M., Adams D.J., Lyon R.M., Takai S. (1991). Tensile properties of the human femur-anterior cruciate ligament-tibia complex. The effects of specimen age and orientation. Am. J. Sports Med..

[48] Mont M.A., Elmallah R.K., Cherian J.J., Banerjee S., Kapadia B.H. (2016). Histopathological Evaluation of the Anterior Cruciate Ligament in Patients Undergoing Primary Total Knee Arthroplasty. J. Arthroplasty.

[49] Johnson A.J., Howell S.M., Costa C.R., Mont M.A. (2013). The ACL in the arthritic knee: How often is it present and can preoperative tests predict its presence?. Clin. Orthop. Relat. Res..

[50] Ishii Y., Noguchi H., Sato J., Yamamoto T., Takayama S., Toyabe S. (2016). Macroscopic evaluation of the anterior cruciate ligament in osteoarthritic patients undergoing total knee arthroplasty. Eur. J. Orthop. Surg. Traumatol..

[51] Berni M., Marchiori G., Cassiolas G., Grassi A., Zaffagnini S., Fini M., Lopomo N.F., Maglio M. (2021). Anisotropy and inhomogeneity of permeability and fibrous network response in the pars intermedia of the human lateral meniscus. Acta Biomater..

